# Childhood‐Onset Takayasu Arteritis With Extensive Vascular Involvement: A Four‐Decade Follow‐Up Case Report

**DOI:** 10.1155/crrh/6725765

**Published:** 2026-08-28

**Authors:** Naylya Djumaeva, Gulnara Achundjanova, Leyla Djumaeva

**Affiliations:** ^1^ Research Institute of Virology of the Republican Specialized Scientific and Practical Medical Center of Epidemiology, Microbiology, Infectious and Parasitic Diseases, Tashkent, Uzbekistan; ^2^ “Avicenna” Medical Center, Tashkent, Uzbekistan

**Keywords:** case report, childhood-onset, collateral circulation, long-term follow-up, Takayasu arteritis, vascular reconstruction

## Abstract

Childhood‐onset Takayasu arteritis is rare, and data on outcomes beyond two or 3 decades are exceptionally limited. We report one of the longest documented follow‐up observations of childhood‐onset Takayasu arteritis, spanning more than 4 decades. The disease began at 9 years of age, and the patient was diagnosed with morphologically confirmed Type III Takayasu arteritis at the age of 16 years. She underwent renal vascular angioplasty and prosthetic reconstruction of the abdominal aorta for left renal artery occlusion associated with severe renovascular hypertension. In 2010, progression of supraaortic vascular disease required aortocarotid bypass surgery, followed six months later by left nephrectomy for persistent severe hypertension. Long‐term medical management included individualized intermittent treatment with low‐dose prednisolone and methotrexate. Despite extensive vascular involvement, the clinical course was characterized by gradual stabilization, progressively longer periods of clinical stability, and preservation of functional independence. Recent laboratory findings demonstrated only mild elevations in inflammatory markers, while vascular imaging showed chronic extensive arterial lesions with well‐developed collateral circulation, preserved cardiac function, and preserved function of the solitary right kidney. The favorable long‐term course was likely multifactorial, reflecting staged vascular reconstruction, individualized medical management, and adaptive collateral circulation. This case provides rare insight into the long‐term evolution and management of severe childhood‐onset Takayasu arteritis.

## 1. Introduction

Takayasu arteritis (TA) is a rare chronic granulomatous large‐vessel vasculitis affecting the aorta and its major branches. The disease predominantly affects young women and may lead to progressive arterial stenosis, occlusion, aneurysm formation, and ischemic complications [1, 2]. Despite advances in diagnosis and treatment, its long‐term clinical and vascular evolution remains incompletely characterized because prolonged longitudinal observations are uncommon.

Although long‐term survival has improved substantially, published reports extending beyond three to 4 decades after disease onset remain exceptionally rare, particularly in patients with childhood‐onset disease. Consequently, the long‐term evolution of advanced vascular lesions, collateral circulation, outcomes of staged vascular reconstruction, and preservation of functional status has not been sufficiently documented.

We report a patient with childhood‐onset TA followed for more than 4 decades after disease onset, including long‐term surgical outcomes, vascular adaptation, collateral vessel development, and preservation of functional status over more than 4 decades. During the course of the disease, she underwent staged major vascular reconstructive procedures and subsequent nephrectomy. This prolonged observation provides a rare opportunity to document the evolution of severe childhood‐onset disease from childhood to late adulthood, including long‐term surgical outcomes, vascular adaptation, collateral vessel development, and individualized medical management.

## 2. Case Presentation

This patient was previously described in two publications addressing distinct stages of her disease course. The first report, published in 1988, documented the initial presentation, early diagnostic difficulties, and subsequent confirmation of TA [3]. A subsequent publication described the intermediate clinical course and treatment history [4]. The present report extends these earlier observations by providing substantially longer follow‐up, updated vascular findings, subsequent surgical interventions, current treatment status, and long‐term functional outcome.

In 1979, a 9‐year‐old female patient developed fever, musculoskeletal pain, and an elevated erythrocyte sedimentation rate approximately six weeks after rabies vaccination. She was initially diagnosed with ankylosing spondylitis; however, this diagnosis was subsequently reconsidered following the development of severe arterial hypertension and renal vascular involvement.

At the age of 16 years, angiographic assessment demonstrated abdominal aortic involvement and left renal artery occlusion associated with severe renovascular hypertension. Type III TA was diagnosed and confirmed by a morphological examination of vascular tissue. The patient underwent renal vascular angioplasty and prosthetic reconstruction of the abdominal aorta at the Vishnevsky Institute of Surgery in Moscow [3].

From 1985 to 2000, the patient remained under long‐term follow‐up at the Vishnevsky Institute of Surgery in Moscow. During this period, her clinical condition remained relatively stable, although chronic arterial hypertension persisted and required continuous antihypertensive therapy. In 1992, she experienced clinical deterioration associated with the progression of vascular disease; however, conservative management was continued, and no additional vascular intervention was performed.

In 2001, at the age of 31 years, the patient was referred to our clinic for further evaluation and long‐term follow‐up. At presentation, she had symptomatic advanced large‐vessel disease manifested by constitutional symptoms, recurrent dizziness and syncope, upper‐limb claudication, paroxysmal tachycardia, and progressive weight loss. Clinical examination confirmed severe impairment of upper‐extremity arterial circulation consistent with advanced TA.

At the initial stage of management in our clinic, the patient received two short courses of adjunctive complex homeopathic preparations. The limited evidence supporting this intervention in TA is addressed separately in the Discussion. Shortly thereafter, individualized low‐dose intermittent prednisolone therapy was initiated and was subsequently used during periods of clinical deterioration.

Despite initial clinical improvement, the progression of supraaortic vascular disease eventually required further surgical intervention. In 2010, the patient underwent aortocarotid bypass surgery at the Republican Specialized Surgical Center named after Academician V. Vakhidov in Tashkent, Uzbekistan. Approximately 6 months later, left nephrectomy was performed because of persistent severe renovascular hypertension.

Following the 2010 surgical interventions, methotrexate was recommended by the surgical team at a dose of 20 mg/week; however, this dose was not used. The maximum dose actually administered was 3.75 mg/week, which was subsequently reduced to 2.5–0.625 mg/week. Prednisolone was administered intermittently at individualized doses ranging from 10 to 1 mg/day, with an average dose of approximately 5 mg/day. Courses of prednisolone and methotrexate were generally limited to periods of clinical deterioration, usually lasted no longer than 2 months, and were discontinued after clinical stabilization. Exacerbations were most often observed in association with upper respiratory or urinary tract infections. At the most recent follow‐up, the patient was no longer receiving prednisolone or methotrexate.

Over subsequent years, the patient demonstrated gradual clinical stabilization, with progressively longer intervals without clinically significant deterioration. Constitutional symptoms, upper‐limb pain, recurrent syncope, dizziness, and episodes of paroxysmal tachycardia resolved, while body weight and physical capacity improved. Despite the persistence of extensive chronic vascular lesions, she remained independent in daily life and maintained active social participation. At the most recent follow‐up, at the age of 57 years, she was clinically stable without glucocorticoid or methotrexate therapy. The principal clinical events, interventions, and outcomes over more than 4 decades of follow‐up are summarized in Table 1.

**TABLE 1:** Clinical timeline of Takayasu arteritis.

Year	Age	Clinical event	Intervention and outcome
1979	9	Antirabies vaccination followed by onset of the first symptoms	Temporal association recorded; causality cannot be established
December 1985	16	Type III Takayasu arteritis diagnosed and morphologically confirmed; left renal artery occlusion and severe renovascular hypertension	Renal vascular angioplasty and abdominal aortic prosthetic reconstruction at the Vishnevsky Institute of Surgery, Moscow
1985–2000	16–31	Follow‐up at the Moscow Institute of Surgery	Long‐term specialist follow‐up; persistent arterial hypertension requiring continuous antihypertensive therapy; clinical deterioration documented in 1992, without additional vascular intervention.
January 2001	31	First evaluation at the authors’ clinic	Weakness, fatigue, dizziness during head rotation, syncope, headache, paroxysmal tachycardia, bilateral upper‐limb exertional pain, and progressive weight loss
2001	31	Initial adjunctive treatment	Two short courses of adjunctive complex homeopathic preparations; their independent contribution to the subsequent clinical course cannot be determined.
2001–2009	31–40	Long‐term clinical management	Intermittent low‐dose prednisolone, usually 1–10 mg/day during exacerbations
January 2010	40	Progression of supra‐aortic vascular disease	Aortic–carotid bypass at the Vakhidov Surgical Center, Tashkent
2010	40–41	Methotrexate introduced	Maximum 3.75 mg/week
Approximately 6 months after bypass	41	Persistent severe arterial hypertension	Left nephrectomy
2010–2 March 2026	41–56	Long‐term individualized management	Intermittent prednisolone and methotrexate courses until 2 March 2026; continuous antihypertensive, antiplatelet, lipid‐lowering, and supportive therapy.
2019–2025	50–56	Increasing clinical stability	Intervals without clinically significant deterioration became progressively longer.
2024–2025	55–56	Recent laboratory and vascular assessment	Mild or borderline inflammatory‐marker abnormalities; chronic extensive vascular lesions with compensatory collateral circulation
Present	57	Current clinical stability	Preserved independent functioning and active professional practice; no prednisolone or methotrexate since 2 March 2026

### 2.1. Laboratory Findings

Laboratory findings evolved over the course of follow‐up. During the earlier decades of the disease, repeated laboratory assessments performed during hospitalizations, surgical interventions, and routine clinical monitoring demonstrated persistent inflammatory abnormalities, which were more pronounced during periods of clinical deterioration.

In recent years, inflammatory activity gradually declined, and investigations performed between 2022 and 2025 showed only mild elevations of inflammatory markers. Hematological, renal, hepatic, and metabolic parameters remained generally stable, while lipid levels were regularly monitored and treated as part of long‐term cardiovascular risk management. Representative laboratory findings from the most recent years are summarized in Table 2.

**TABLE 2:** Representative laboratory investigations during recent follow‐up (2022–2025).

Parameter	Date	Result	Laboratory reference range	Interpretation
ESR	5 Jun 2025	29 mm/h	< 30 mm/h	Within laboratory range
ESR	28 Nov 2025	16.39 mm/h	2–15 mm/h	Slightly elevated
CRP	28 Nov 2025	10.6 mg/L	0–10 mg/L	Slightly elevated
D‐dimer	30 Jul 2022	282 ng/mL	< 243 ng/mL	Slightly elevated
D‐dimer	28 Nov 2025	0.876 mg FEU/mL	< 0.5 mg FEU/mL	Elevated
Fibrinogen	28 Nov 2025	4.36 g/L	2.76–4.71 g/L	Within laboratory range
Hemoglobin	5 Jun–28 Nov 2025	128–140 g/L	117–160 g/L	Normal
Leukocytes	5 Jun–28 Nov 2025	6.57–6.98 × 10^9^/L	4.0–11.0 × 10^9^/L	Normal
Platelets	5 Jun–28 Nov 2025	235–244 × 10^9^/L	150–400 × 10^9^/L	Normal
Creatinine	2 Jul 2024	49 μmol/L	49–90 μmol/L	Normal
Creatinine	28 Nov 2025	47.2 μmol/L	44–88 μmol/L	Normal
ALT	2 Jul 2024	26 U/L	0–35 U/L	Normal
ALT	28 Nov 2025	21 U/L	0–35 U/L	Normal
AST	2 Jul 2024	22 U/L	< 31 U/L	Normal
AST	28 Nov 2025	19 U/L	14–36 U/L	Normal
Total cholesterol	2 Jul 2024	6.07 mmol/L	< 5.0–5.2 mmol/L	Elevated
LDL‐C	2 Jul 2024	3.67 mmol/L	Individual target < 3.0 mmol/L	Elevated
Triglycerides	2 Jul 2024	2.06 mmol/L	< 1.7 mmol/L	Elevated
Total cholesterol	28 Nov 2025	4.2 mmol/L	< 5.2 mmol/L	Normal
LDL‐C	28 Nov 2025	2.05 mmol/L	0.08–2.59 mmol/L	Within laboratory range
Triglycerides	28 Nov 2025	1.21 mmol/L	0.39–1.52 mmol/L	Normal
Fasting insulin	2 Jul 2024	12.5 µU/mL	2.7–10.4 µU/mL	Slightly elevated
Fasting insulin	28 Nov 2025	13.8 µU/mL	2.3–24.8 µU/mL	Normal
HbA1c	28 Nov 2025	5.3%	4.0%–6.5%	Normal
Ferritin	2 Jul 2024	26 ng/mL	15–204 ng/mL	Normal
Ferritin	28 Nov 2025	20.3 ng/mL	15–150 ng/mL	Low‐normal
25‐OH vitamin D	28 Nov 2025	75.5 ng/mL	30–100 ng/mL	Normal
Rheumatoid factor	28 Nov 2025	< 8.6 IU/mL	< 12 IU/mL	Negative
ASL‐O	12 Aug 2022	< 50 IU/mL	< 200 IU/mL	Negative

*Note:* D‐dimer results are reported in the original laboratory units because different assays and reference intervals were used; direct numerical comparison between the 2022 and 2025 values is therefore inappropriate.

### 2.2. Vascular Findings

Vascular imaging and surgical records documented extensive chronic large‐vessel involvement and the long‐term consequences of staged vascular reconstruction. The principal findings included chronic occlusion of the left common carotid artery, severe stenosis of the left subclavian artery with retrograde vertebral flow, lesser right‐sided arterial stenoses, and well‐developed collateral circulation. The aortocarotid bypass and abdominal aortic prosthesis remained patent, while perfusion of the solitary right kidney was preserved. Echocardiography showed mild dilation of the ascending aorta and aortic sinuses, mild aortic regurgitation, preserved left ventricular systolic function, and normal pulmonary artery pressure. The major vascular interventions and imaging findings are summarized in Table 3.

**TABLE 3:** Major vascular interventions and imaging findings during follow‐up.

Year	Procedure or imaging modality	Main findings
1985	Angiography, surgical assessment, and morphological confirmation	Angiographic assessment demonstrated abdominal aortic involvement and left renal artery occlusion associated with severe renovascular hypertension

1985	Vascular surgery	Renal vascular angioplasty and prosthetic reconstruction of the abdominal aorta. The abdominal aortic prosthesis measured approximately 15 mm in diameter; renal vascular angioplasty and prosthetic reconstruction of the abdominal aorta. The right renal artery measured approximately 5‐6 mm, with no hemodynamically significant stenosis reported at that time

January 2010	Vascular surgery	Aortic–carotid bypass for progressive supraaortic vascular disease

2010	Surgery	Left nephrectomy for persistent severe renovascular hypertension

15 Nov 2024	Duplex ultrasonography of brachiocephalic arteries	Chronic occlusion of the left common carotid artery; perfusion of distal vessels through shunt and collateral pathways. Distal perfusion was maintained through a patent bypass graft without significant distal anastomotic restenosis
	Duplex ultrasonography	Approximately 80% stenosis of the left subclavian artery with collateral distal flow
	Vertebral artery Doppler	Retrograde flow in the left vertebral artery, consistent with left subclavian steal syndrome
	Duplex ultrasonography	Approximately 30% stenotic changes in the right common carotid bifurcation and right subclavian artery
	Duplex ultrasonography	Well‐developed collateral circulation providing hemodynamic compensation
	Renal and aortic duplex ultrasonography	The abdominal aortic prosthesis remained patent and measured approximately 15 mm in diameter; no hemodynamically significant stenosis of the right renal artery was detected
	Renal ultrasonography	Solitary right kidney after left nephrectomy; renal perfusion preserved; renal cysts present
	Echocardiography	Mild dilation of the sinuses of Valsalva and ascending aorta; mild aortic regurgitation; preserved left ventricular systolic function, LVEF 58%; normal estimated pulmonary artery pressure

Despite the persistence of extensive chronic vascular lesions, the reconstructed vessels remained functional, collateral circulation was well developed, and end‐organ perfusion was preserved.

## 3. Discussion

Long‐term, multidecade follow‐up of childhood‐onset TA is exceptionally uncommon in the published literature, and it is precisely this scarcity that gives the present observation its value. Most reports describing childhood‐onset TA cover follow‐up intervals of several years to, at most, 1 to 2 decades [5–10], and even large multicenter cohorts addressing long‐term prognosis in TA generally extend to a median of 10 to 15 years [8]. The present patient has now been followed for more than 4 decades, from disease onset at age 9 through diagnosis in adolescence into her sixth decade of life. Such an extended observation window offers a rare opportunity to document the natural evolution of severe vascular disease, the durability of staged surgical reconstruction, and the long‐term trajectory of functional status—information that shorter cohort studies, by design, cannot provide. It is this prolonged observation period, rather than any single therapeutic intervention, that constitutes the principal contribution of the present report.

Childhood‐onset TA represents a minority of all TA cases but is consistently described as differing from adult‐onset disease. Pediatric cohorts report a higher prevalence of abdominal aortic and renal artery involvement, a greater likelihood of hypertension at presentation, and a more aggressive early disease course frequently requiring surgical intervention [5–9]. A recent French multicenter series similarly found that renal artery involvement and the need for revascularization were common in children with TA, while a Mexican national cohort of pediatric‐onset TA reported comparable patterns of severe vascular involvement and early surgical need [9, 10]. This longitudinal case is concordant with these patterns: disease onset at age 9, renal artery occlusion with severe renovascular hypertension identified within the first years of disease, and staged surgical reconstruction beginning in adolescence. What distinguishes this case from most published pediatric cohorts is not the severity of vascular involvement at onset, which is a recognized hallmark of childhood‐onset disease, but the subsequent four‐decade trajectory—a follow‐up duration that existing pediatric series, constrained by their inherently shorter observation periods, cannot describe.

The central finding of this observation is functional rather than pharmacological: despite occlusion of the left common carotid artery, severe subclavian stenosis with steal physiology, aortic root involvement, and loss of one kidney, the patient has maintained social independence throughout her sixth decade of life. Three factors plausibly converged to produce this trajectory. First, staged surgical correction—renal angioplasty and aortic prosthesis in adolescence, followed decades later by aortic‐carotid bypass and nephrectomy—addressed critical stenoses and hypertension as they became hemodynamically significant, rather than being deferred until catastrophic failure occurred. Second, well‐developed collateral circulation, documented on imaging decades after the original vascular insults, likely buffered the hemodynamic consequences of chronic large‐vessel occlusion; collateral vessel formation is a recognized adaptive response to chronic arterial occlusion, and its development is itself modulated by the same chronic inflammatory and immune signaling that drives TA [11–13]. Third, sustained multidecade follow‐up allowed disease progression to be identified and addressed incrementally. None of these three elements is unique to this patient, but their combination, sustained over 40 years, is what this case can credibly illustrate: that severe early vascular involvement in childhood‐onset TA does not preclude a favorable long‐term functional outcome, provided disease progression is surgically corrected as it arises, and the patient remains under adequate long‐term monitoring.

Any discussion of the medical management in this longitudinal case must be read against the fact that treatment began in the 1980s, decades before the publication of the therapeutic frameworks that now guide TA management, including the 2021 American College of Rheumatology/Vasculitis Foundation guideline [14], the French national recommendations [2], and the 2023 EULAR recommendations on the use of imaging in large‐vessel vasculitis [15]. Recent cohort data have also begun to characterize gender‐related clinical patterns and predictors of biologic therapy requirement in TA, which may help contextualize individualized treatment decisions such as those made in the present case [16]. Contemporary management of TA typically combines induction‐phase glucocorticoids with a conventional or biologic steroid‐sparing agent, selected and monitored according to standardized protocols, and imaging‐based activity assessment [1, 14, 15, 17]. The treatment this patient received—including two short courses of adjunctive homeopathic preparations at the outset of her care under the present authors, and subsequently intermittent low‐dose corticosteroids and methotrexate—reflects the therapeutic options and standards of practice available at the time and place of treatment, rather than representing a deliberate deviation from a contemporary standard that did not yet exist when her disease course began. We present this treatment history transparently as an accurate clinical record; it is not intended as a model for present‐day practice, which should continue to follow current ACR/EULAR‐based recommendations.

From 2001 onward, the patient received corticosteroids and, later, methotrexate at doses substantially below those typically reported for induction or maintenance therapy in TA, administered intermittently rather than continuously [1, 17, 18]. We wish to be explicit about the limits of this long‐term follow‐up. It does not demonstrate that low‐dose intermittent therapy is an effective or sufficient strategy for TA, and we do not recommend it as an alternative to standard‐dose induction therapy. The patient’s clinical course cannot be dissociated from the effect of the surgical interventions she underwent, and the relative contributions of pharmacological therapy, surgery, and the natural evolution of disease activity over 4 decades cannot be determined from a single observation. We report the dosing history because it forms part of an accurate clinical record, not because it constitutes evidence supporting an alternative treatment strategy.

The relationship between environmental immune stimulation and the onset of autoimmune vasculitis remains an active area of investigation. Infection has long been proposed as a potential contributor to TA pathogenesis, including a possible role for *Mycobacterium tuberculosis* [19], and broadly, viral and bacterial infections are recognized to be capable of triggering or unmasking autoimmune disease in genetically susceptible individuals through mechanisms that include molecular mimicry and bystander immune activation [20–22]. Vaccination has similarly been discussed as a potential, though unproven, trigger of vasculitis: large‐vessel vasculitis has been reported after hepatitis B vaccination [23], and TA has been reported to develop or relapse following COVID‐19 vaccination [24], within a broader literature of vaccine‐associated vasculitis [25]. Antirabies vaccination specifically has also been associated with vasculitic reactions, including cutaneous small‐vessel vasculitis [26], although an association with large‐vessel vasculitis such as TA has not, to our knowledge, been previously reported. In the present case, the onset of constitutional symptoms approximately 6 weeks after antirabies vaccination at age 9 is a notable temporal association that adds a further data point to this ongoing discussion. We present it explicitly as a clinical observation, not as evidence of causation: a coincidental relationship cannot be excluded, no mechanistic data are available in this patient, and the 6‐year interval between symptom onset and eventual morphological diagnosis further limits any causal inference.

At the initial stage of care under the present authors, the patient received two short courses of adjunctive complex homeopathic preparations before conventional corticosteroid therapy was introduced. We report their use here strictly as a factual component of the treatment timeline [4]. Homeopathy lacks a pharmacological mechanism consistent with established principles of pharmacology, and no controlled clinical evidence supports its efficacy in TA or any other large‐vessel vasculitis. Because these preparations were administered before, and in temporal proximity to, the initiation of corticosteroid therapy, it is not possible to attribute any component of the patient’s subsequent clinical course to the homeopathic intervention, and we draw no conclusions regarding its efficacy.

### 3.1. Strengths and Limitations

This report has notable strengths, foremost among them an observation period exceeding 4 decades—among the longest reported for childhood‐onset TA—encompassing morphologically confirmed diagnosis, staged surgical management, and multidecade functional follow‐up in a single, well‐characterized patient. Its limitations must be weighed accordingly. It describes a single patient, precluding causal or generalizable conclusions. Detailed laboratory data from the 1985–2022 period were not retained in a form suitable for longitudinal analysis, limiting biochemical assessment largely to the most recent years of follow‐up. Vascular imaging was not systematically performed between 1985 and 2010, so disease activity during this interval was inferred from clinical and laboratory monitoring rather than direct vascular assessment. Finally, the sequential use of surgery, corticosteroids, methotrexate, and homeopathic preparations at different points in the disease course precludes attribution of the favorable outcome to any single intervention.

Taken together, this case illustrates not a specific treatment strategy but the natural history of severe childhood‐onset TA followed for nearly half a century. The disease began with aggressive vascular involvement in childhood and subsequently evolved through staged surgical correction, adaptive collateral circulation, individualized medical management, and prolonged clinical surveillance. Despite extensive irreversible vascular damage, functional independence was preserved. The interventions described are components of this longitudinal history rather than evidence supporting the efficacy of any single therapeutic approach.

Exceptionally prolonged longitudinal observations such as the present case complement cohort studies by providing insights into disease evolution that cannot be captured within conventional follow‐up periods and may help refine expectations regarding long‐term functional outcome in childhood‐onset TA.

## Funding

The study was conducted without external funding or grants.

## Ethics Statement

Written informed consent was obtained from the patient for publication of all clinical data presented in this case report, including laboratory and imaging findings.

Ethical approval was not required for this study in accordance with local regulations and institutional guidelines for single case reports.

## Conflicts of Interest

The authors declare no conflicts of interest.

## Supporting Information

Additional supporting information can be found online in the Supporting Information section.

## Supporting information


**Supporting Information** CARE‐checklist.

## Data Availability

The clinical data supporting the findings of this case report are not publicly available because of patient privacy and ethical considerations. Previously published reports describing the earlier clinical course of this patient are publicly available in the Zenodo repository (https://zenodo.org/records/20007773; DOI: 10.5281/zenodo.20007773).
