# Supplementary material for: Childhood‐Onset Takayasu Arteritis With Extensive Vascular Involvement: A Four‐Decade Follow‐Up Case Report
Source: Case Rep Rheumatol. 2026 Aug 28;2026:6725765. doi: 10.1155/crrh/6725765 (PMC13522926; doi:10.1155/crrh/6725765)
Supplement: Supplementary file 1 [file CRRH-2026-6725765-s001.pdf]

CARE case report guidelines

CARE Checklist of information to include when writing a case report

(cc) BY-NC-ND

Topic	Item	Checklist item description	Reported on Line
Title	1	The diagnosis or intervention of primary focus followed by the words "case report"	1-2
Key Words	2	2 to 5 key words that identify diagnoses or interventions in this case report, including "case report"	30-31
Abstract (no references)	3a	Introduction: What is unique about this case and what does it add to the scientific literature?	11-13,26-29
	3b	Main symptoms and/or important clinical findings	14-26
	3c	The main diagnoses, therapeutic interventions, and outcomes	13-14
	3d	Conclusion—What is the main "take-away" lesson(s) from this case?	26-29
Introduction	4	One or two paragraphs summarizing why this case is unique (**may include references**)	44-45,48-52
Patient Information	5a	De-identified patient specific information	44
	5b	Primary concerns and symptoms of the patient	61-65,78-82
	5c	Medical, family, and psycho-social history including relevant genetic information	66-76
	5d	Relevant past interventions with outcomes	69-76,88-92
Clinical Findings	6	Describe significant physical examination (PE) and important clinical findings	80-82
Timeline	7	Historical and current information from this episode of care organized as a timeline	61-82,88-92
Diagnostic Assessment	8a	Diagnostic testing (such as PE, laboratory testing, imaging, surveys).	121,131
	8b	Diagnostic challenges (such as access to testing, financial, or cultural)	n/a
	8c	Diagnosis (including other diagnoses considered)	55-56,63
	8d	Prognosis (such as staging in oncology) where applicable	105-109
Therapeutic Intervention	9a	Types of therapeutic intervention (such as pharmacologic, surgical, preventive, self-care)	66-70,73-74,83-87,89-93
	9b	Administration of therapeutic intervention (such as dosage, strength, duration)	93-99
	9c	Changes in therapeutic intervention (with rationale)	n/a
Follow-up and Outcomes	10a	Clinician and patient-assessed outcomes (if available)	102-105
	10b	Important follow-up diagnostic and other test results	112-117
	10c	Intervention adherence and tolerability (How was this assessed?)	97-99
	10d	Adverse and unanticipated events	n/a
Discussion	11a	A scientific discussion of the strengths AND limitations associated with this case report	241-252
	11b	Discussion of the relevant medical literature **with references**	136-240
	11c	The scientific rationale for any conclusions (including assessment of possible causes)	253-263
	11d	The primary "take-away" lessons of this case report (without references) in a one paragraph conclusion	260-263
Patient Perspective	12	The patient should share their perspective in one to two paragraphs on the treatment(s) they received	co-author of the article
Informed Consent	13	Did the patient give informed consent? Please provide if requested	Yes +☐ No ☐
